# Static and Fatigue Behavior of Rubber-Sleeved Stud Shear Connectors as Part of Field-Cast Ultra-High Performance Concrete Connections

**DOI:** 10.3390/ma13102269

**Published:** 2020-05-14

**Authors:** Zhigang Zhang, Xiaoqing Xu

**Affiliations:** 1 School of Civil Engineering, Chongqing University, Chongqing 400045, China; zhangzg@cqu.edu.cn; 2 Key Laboratory of New Technology for Construction of Cities in Mountain Area (Chongqing University), Ministry of Education, Chongqing 400045, China

**Keywords:** ultra-high performance concrete, rubber, rubber-sleeved stud, fatigue, shear connector, ductility, headed stud, precast concrete, accelerated bridge construction

## Abstract

Field-cast ultra-high performance concrete (UHPC) connections are an innovative and prospective solution for combining full-depth precast concrete decks and steel girders. However, previous studies show that the slip capacity of stud shear connectors embedded in UHPC cannot meet the requirements for ductile connectors by Eurocode 4, which can reduce the resistance of steel and concrete composite members. In this study, the rubber-sleeved stud shear connector, which is a composite of ordinary stud and rubber sleeve, was adopted for the field-cast UHPC connections. Push-out tests were conducted to investigate the static and fatigue behavior of the rubber-sleeved stud shear connector as part of field-cast UHPC connections. Results of static tests showed that the rubber-sleeved stud shear connector has sufficient deformation capacity and its slip capacity is 1.5 times that of the ordinary stud shear connector. Compared to ordinary stud shear connectors, UHPC with high strength and stiffness has a relatively small effect on improving the shear strength and stiffness of rubber-sleeve stud shear connectors. Results of fatigue tests showed that the rubber-sleeved stud shear connector in UHPC has similar fatigue behavior to that in normal strength concrete. Though UHPC improves the restraint to the stud deformation, the influence of rubber sleeves is still decisive in determining the fatigue behavior of rubber-sleeve stud shear connectors. In addition, based on the results of strain gauges at stud roots, it was found that the crack initiation process consumes a small proportion of the fatigue life of rubber-sleeved stud shear connectors, which is about 5%.

## 1. Introduction

The full-depth precast concrete deck system is one of the promising technologies for accelerated bridge construction (ABC) of steel and concrete composite bridges [1,2]. It needs no framework in place and significantly accelerates the construction of bridges, and dramatically minimize delays and disruptions to the community [3]. Moreover, the system significantly improves the quality control of concrete and decreases life-cycle costs.

**Figure 1:**
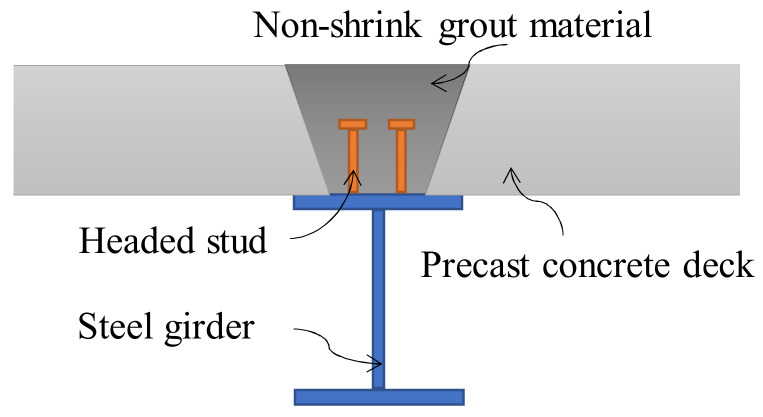
Steel and concrete composite beam with precast decks.

For steel and concrete composite bridges with precast decks, full-depth voids (termed shear pockets) are reserved on the precast decks. After placing the pockets around shear connectors on the steel girders and grouting the shear pockets, the connection between steel girders and concrete decks is completed. The usual approach is to use non-shrinkable mortar and stud shear connectors, as shown in Figure 1 [4]. 

High concentrated loads are exerted from shear connectors on the filling material. As a result, surface cracking in shear pockets under complex stress state in service is a durability concern. The cracks accelerate the penetration of aggressive agents, resulting in durability reduction [5,6]. Moreover, the properties of the filling material significantly affect the behavior of shear connectors. Premature failure of the filling material before the connectors develop their full strength has to be prevented, especially in case that cracks appear in the shear pockets [7]. Therefore, it is of importance to increase the strength and durability of the filling material.

Ultra-high performance concrete (UHPC) is composed of compact cementitious matrix combined with a high amount of fibers [8] which help to achieve superior mechanical performance and excellent durability of concrete [9,10]. Owing to these advantages, it has been applied in the construction of bridges all over the world in recent years [11]. One of the competitive applications is the field-cast UHPC connections. The field-cast UHPC engages discrete connectors both in the deck and on the girder and, through its high sustained tensile capacity, transfers applied forces between the prefabricated components [12,13]. Researches have shown that the field-cast UHPC connections are capable of meeting critical design, construction, and response requirements [14,15,16]. Moreover, the dimensions and complexity of the joint are significantly reduced, and the speed of construction is improved. In this study, the behavior of stud shear connectors as part of the field-cast UHPC connections was investigated.

Researches on stud shear connectors as part of the field-cast UHPC connections for steel and concrete composite bridges are lacking so far. In 2018, Wang et al. [17] proposed a novel steel-UHPC composite system, and UHPCs were used as the filling material for the shear pocket on precast normal strength concrete (NSC) slabs. Push-out tests on stud shear connectors of 30mm diameter in the field-cast UHPC connection were conducted. It was found that UHPC could limit the crack growth in precast NSC slabs, and the use of UHPC could reduce the construction time and costs. In addition to this study, many researchers used UHPC as the material for the thin bridge slab deck, and the behavior of short stud shear connectors in thin UHPC slabs was investigated. Dieng et al. [18] conducted push-out tests on the steel-UHPC connections with studs, and observed that the failure of the connection was occurred by the yielding of the stud toe. The study by Kim et al. [19] investigated the stud shear connectors embedded in thin UHPC slabs. It was shown the average slip capacity of stud shear connectors was smaller than the required slip capacity, 6 mm. The performance of studs embedded in steel-fiber-reinforced cementitious composites (SFRCC), a type of UHPC, was investigated by Luo et al. [20]. A fiber volume fraction of 6% was found to be most suitable to ensure the ductility of the shear connectors. Cao et al. [21] studied the static and fatigue behavior of short studs embedded in UHPC. It was revealed that weld collars contribute to a significant portion of the shear strength and should be taken into account in the strength prediction for studs in UHPC. The proposed shear stress range vs. fatigue life curve (S-N curve) for studs in UHPC lies slightly above the S-N curve specified in Eurocode 4 [22] for studs in NSC. Wang et al. [23] reported the results of push-out tests on demountable headed stud shear connectors in steel-UHPC composite structures. It was found that though the ductility of the demountable shear connectors is superior to conventional welded ones, it is still less than the requirements for ductile shear connectors in Eurocode 4. In addition, the numerical and experimental studies by Qi et al. [24] indicated that the shear capacity of studs in UHPC is supposed to be composed of two parts, stud shank shear contribution and concrete wedge block shear contribution.

**Figure 2:**
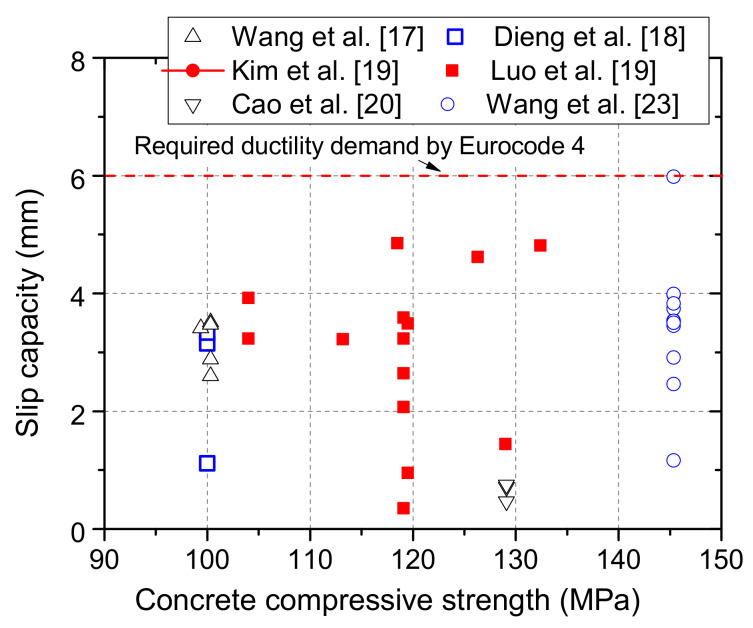
Slip capacities of stud shear connectors in ultra-high performance concrete (UHPC).

Results of the value of slip capacity, the maximum slip at the load which is 10% less than the peak load [22], from the above studies show that the ductility of stud shear connectors embedded in UHPC cannot meet the requirements for ductile shear connectors in Eurocode 4, as shown in Figure 2. 

Local connection shear failure in the absence of adequate connection ductility can reduce steel and concrete composite element bending resistance [25]. Therefore, when using UHPC as the filling material, the ductility of shear connectors needs to be improved.

**Figure 3:**
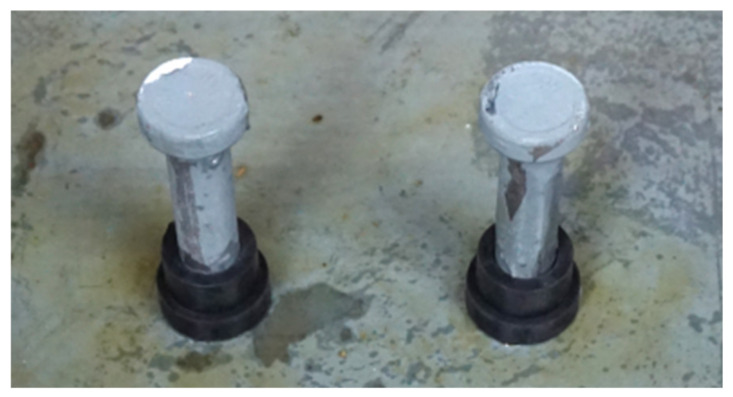
Rubber-sleeved studs.

Rubber-sleeved studs are the studs wrapped with rubber sleeves [26], as shown in Figure 3. 

Rubber has lower stiffness and higher ductility than concrete. As a result, studies by Xu et al. [27] have shown that rubber-sleeved stud shear connectors have higher maximum slip capacities than ordinary stud shear connectors. Therefore, they can be used in UHPC to improve the connection ductility. Although the effect of rubber sleeves on the static and fatigue behavior of the shear connectors in NSC has been clarified [26,27,28,29,30], there is no report on the behavior of rubber-sleeved stud shear connectors embedded in UHPC.

In this paper, static and fatigue behavior of rubber-sleeved stud shear connectors as part of field-cast UHPC connections was studied. Five push-out specimens, including two specimens subjected to static load and three specimens subjected to fatigue load, with precast NSC slabs and field-cast UHPC were fabricated. Through the static tests, the shear strength, shear stiffness, and ductility of rubber-sleeved stud shear connectors in UHPC were studied. Based on the fatigue test results, the fatigue strength curve was investigated and the deformation behavior of the connectors was described and analyzed.

## 2. Experiment Description

### 2.1. Push-Out Test Program

#### 2.1.1. Test Specimens

A total of five push-out specimens, including two specimens subjected to static shear load and three specimens subjected to fatigue shear load, were fabricated and tested. The details of the specimens are summarized in Table 1. 

**Figure 4:**
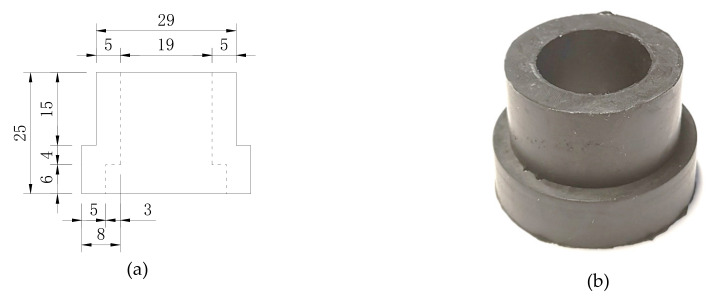
Rubber sleeve: (**a**) details (mm); (**b**) photo.

Headed studs of 19 mm diameter (*d*_s_) and 100 mm height (*h*_s_) are used in all specimens, and the rubber sleeves (Figure 4) are 5 mm in thickness and 25 mm in height. The specimen label was designated as “connector type-shear stress range-concrete type”. ‘‘S’’ and ‘‘RS25’’ stand for ordinary studs and rubber-sleeved studs, respectively. ‘‘U’’ refers to UHPC. The load values (*V*_min_, *V*_max_, Δ*V*) in the table refer to the load applied to one shear connector, and *τ* is the nominal shear stress in the stud.

**Figure 5:**
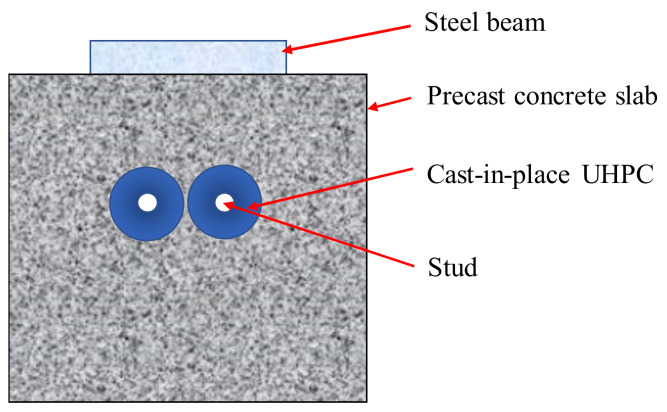
Schematic representation of the specimen.

As shown in Figure 5, the specimen consists of three parts: precast concrete slabs with shear pockets, cast-in-place UHPC, and a steel member with studs. 

**Figure 6:**
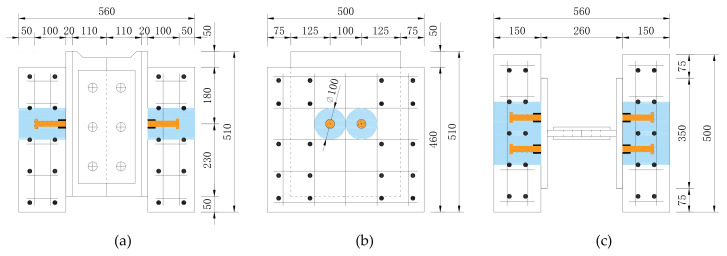
Details of specimens (mm): (**a**) front view; (**b**) side view; (**c**) plan view.

Figure 6 presents the details of the specimens. In the precast concrete slabs, four layers of *ϕ*14 mm steel rebars were placed and two full-depth voids of 10 cm diameter were made for casting UHPC.

#### 2.1.2. Test Setup

**Figure 7:**
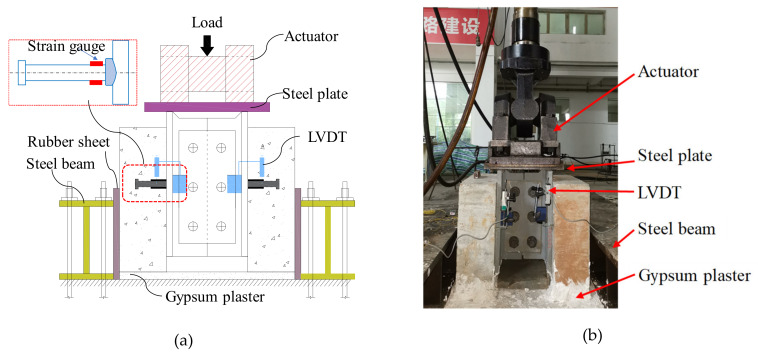
Test setup: (**a**) schematic diagram; (**b**) photo.

The test setup is shown in Figure 7. Push-out test method was adopted [30]. 

A 30 mm thick steel plate was used to distribute the load to the flanges of the steel component of the specimen, so that the shear connectors were under the pure shear load. The relative slips between the concrete slab and the steel component were measured by linear variable displacement transformers (LVDTs) which were attached on the steel flanges. In addition, 5 mm strain gauges were attached to the upper and lower sides of the stud root, and the distance from the center of the strain gauge to the steel beam surface is about 15 mm.

#### 2.1.3. Loading and Measurement Scheme

For the static push-out tests, both displacement control and force control were used. After several loading cycles, a monotonically increasing static load was applied to the specimen until complete failure. The displacement control was used in the failure stage of specimens when significant reduction of the stiffness was observed. The loading speed was controlled under 10 kN/min or 1 mm/min.

**Figure 8:**
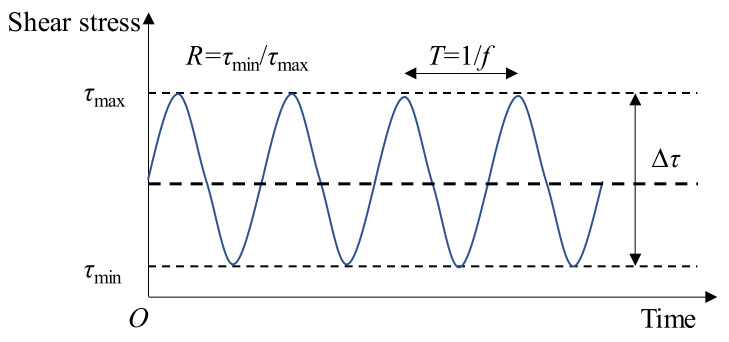
Main parameters in fatigue loading.

For the fatigue push-out tests, force control was used. The main parameters in fatigue loading are shown in Figure 8. The fatigue loading was carried out using a continuous sinusoidal waveform with a loading frequency (*f*) of 4 Hz. The maximum and minimum shear stresses (*τ*_max_ and *τ*_min_) were kept constant during the fatigue loading. All the data was collected by a data acquisition system and the data acquisition is at a sampling rate of 200 Hz.

### 2.2. Specimen Fabrication

**Figure 9:**
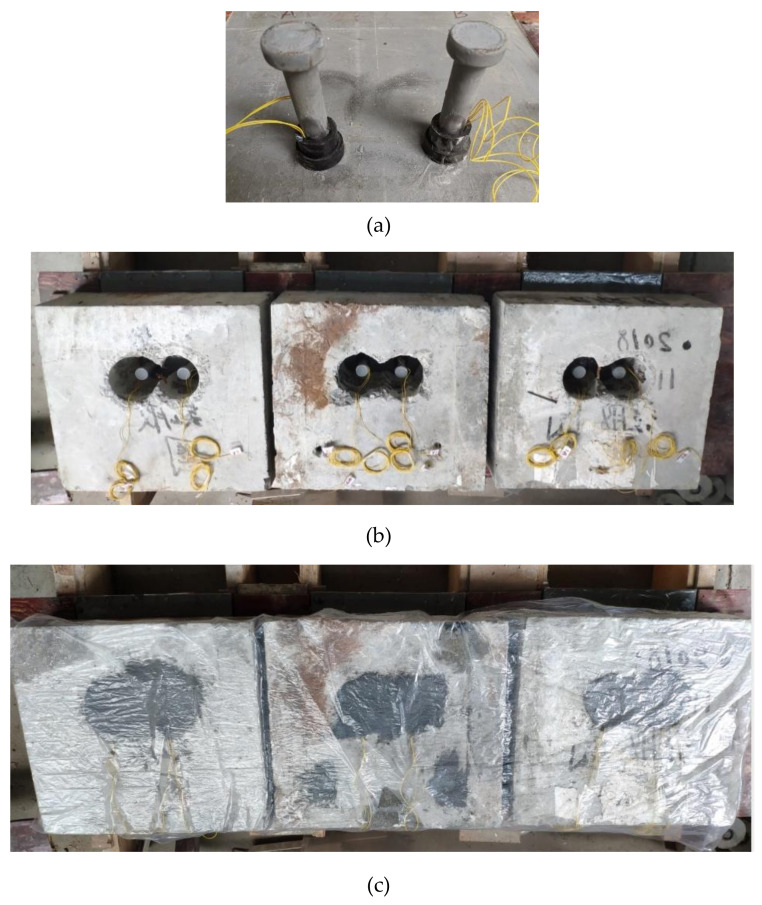
Photos for specimen fabrication: (**a**) strain gauge and rubber sleeve installation; (**b**) placing the precast concrete slab; (**c**) UHPC curing.

The strain gauges were attached to the studs after the studs were welded onto the steel plates. Then, the rubber sleeves were cut, opened and bonded to the studs by the super glue, as shown in Figure 9. The NSC slabs were cast and the full-depth voids were made. Then, the precast concrete slabs were placed on the steel beams. Adhesive tape was applied to the steel beam surface of all specimens to eliminate the influence of adhesion and friction between UHPC and steel beams. Finally, the UHPC was poured, and the specimens were cured in a natural environment.

### 2.3. Material Properties

The mix proportion of NSC for precast concrete slabs is listed in Table 2. Table 3 shows the material properties of NSC. The concrete compressive strength was obtained from compression tests on the 150 mm cube and the 150 mm × 150 mm × 300 mm prism specimens, and tensile splitting strength was obtained by tensile splitting tests of cube specimens. 

**Figure 10:**
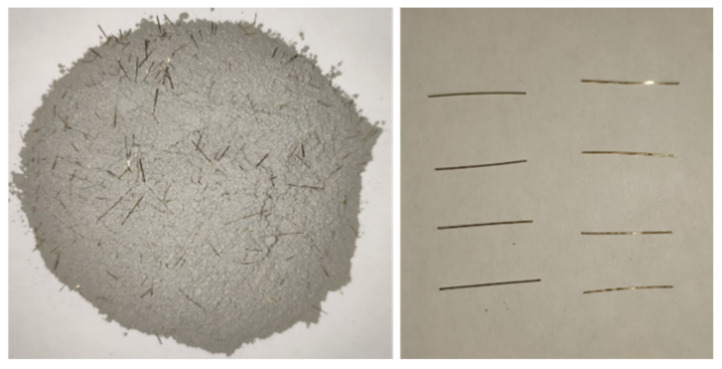
UHPC premix and straight steel fibers.

The UHPC material was composed of water and a commercially available UHPC premix which includes 2% volume fraction of straight, smooth 12-mm-long by 0.2-mm-diameter fibers as shown in Figure 10. The water-binder ratio was 0.18. The compressive strength of UHPC was 124.8 MPa, which was obtained through compressive testing of six 100 mm × 100 mm × 100 mm cubic specimens. Therefore, the concrete compressive strength meets the requirements in the recommendations of the Chinese code for UHPC [31]. 

The rubber sleeves were made of NR45° natural rubber with the hardness of 45 (Shore A). Table 4 shows the material properties of the rubber.

## 3. Static Behavior 

### 3.1. Load–Slip Curve

**Figure 11:**
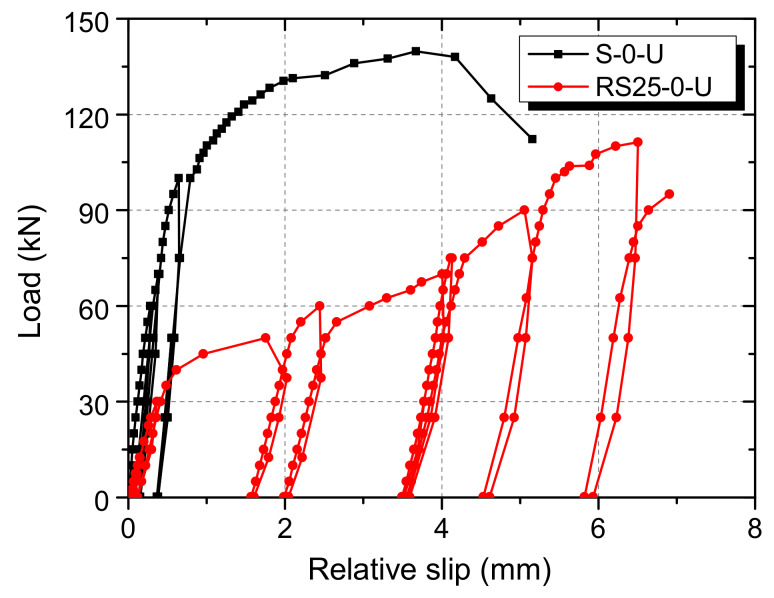
The load-slip curves of static push-out test specimens.

The average slip from four LVDTs is plotted against the load per shear connector, as shown in Figure 11. For the S-0-U specimen, the load-slip curve is generally linear when the load is small. After the load increases to about 100 kN, the shear connector gradually yields, and the slip growth rate increases. As the specimen approaches failure, the slope of the curve turns to be very small. The load-slip curve of the RS25-0-U specimen has high stiffness when the load is under about 40 kN. After that, the slope of the curve decreases rapidly and remains basically unchanged. As the slip continues to increase to about 5 mm, which is equal to the thickness of the rubber sleeve, the slope of the curve increases slightly.

The slip capacities of the S-0-U and RS25-0-U specimens according to Eurocode 4 are 4.6 mm and 6.9 mm, respectively. Although the shear strength of the RS25-0-U specimen is less than that of the S-0-U specimen, the slip capacity is 1.5 times that of the S-0-U specimen. It can be concluded that rubber sleeves effectively improve the ductility of the shear connector in UHPC.

### 3.2. Shear Strength

The shear strengths of the S-0-U and RS25-0-U specimens are 139.8 kN and 111.3 kN, respectively. The shear strength of the rubber-sleeve stud shear connector in UHPC is 79.6% of that of the ordinary stud shear connector. This is different from the test results of the shear connectors in NSC. The study by Xu et al. [27] on the shear connectors in C40 concrete shows that the shear strength of the ordinary stud shear connector is 107.2 kN while that of rubber-sleeve stud shear connector is 99.0 kN. Although both types of shear connectors have larger shear strength in UHPC than that in NSC, UHPC has a relatively small effect on improving the shear strength of rubber-sleeve stud shear connectors.

The existing calculation formulas in design codes for shear strength were developed from the results of tests on studs in NSC. At the same time, there are few studies on studs in high strength concrete such as UHPC. An and Cederwall [32] reported that the shear strength of studs in high strength concrete was underestimated if formulas for studs in NSC was used. The reason is that in these formulas, the shear strength of the stud was determined separately by the concrete or by the stud, and the interaction between concrete and studs was suggested to be taken into account. With increasing concrete strength, the probability is increasing that the weld collar governs the stud failure. The weld collar with a slightly larger diameter than the stud shank was thought to enhance the shear strength of studs. In addition, the concrete splitting wedge in front of the weld collar was assumed to imply an unignorable contribution to the shear strength enhancement of studs in high strength concrete [24]. The dimensions of the wedge increase with the sizes of the weld collar. To account for the above bearing contributions of the weld collar, Doinghaus et al. [33] proposed a formula specifically for the shear strength of headed studs embedded in high strength concrete as the following:

(1) $$V_{u}=\left(0.85A_{s}f_{\mathrm{su}}+\eta_{\mathrm{wc}}f_{c}^{’}d_{\mathrm{wc}}l_{\mathrm{wc}}\right)/\gamma_{v}$$

where *V*_u_ is the shear strength of headed stud shear connector; *A*_s_ is the cross section area of stud shank; *f*_su_ is the tensile strength of the stud; *f*_c_’ is the concrete cylinder compressive strength; *η*_wc_ is the empirical correction factor, which is 1.5; *d*_wc_ is the diameter of the weld collar; *l*_wc_ is the height of the weld collar; and *γ*_v_ is the safety factor of 1.25. The tensile strength of the stud was 450 MPa. For the size of the weld collar, the suggested values in the code GB/T 10433 [34] were adopted, and the diameter and the height were taken as 23 mm and 6 mm, respectively. The cubic compressive strength of UHPC was converted to cylinder one by multiplying by a correction factor, 0.8, according to the Chinese code [31]. As a result, *f*_c_’ is taken as 99.9 MPa. Several researchers suggested that the value of 1.5 is not enough for *η*_wc_, and based on push-out test results, they stated that a value of 2.5 is more suitable.

As shown in Table 5, the shear strengths of specimens are listed, and the calculated results of *η*_wc_ through Equation (1) are presented. 

For the S-0-U specimen, the value of *η*_wc_ was calculated to be 2.27, which is close to 2.5. Therefore, when calculating the shear strength of an ordinary stud shear connector in UHPC, the influence of the welded collar cannot be ignored. However, the value of *η*_wc_ was calculated to be only 0.21 for the RS25-0-U specimen. Moreover, if *f*_c_’ and *η*_wc_ in Equation (1) were taken as 40.0 MPa and zero, respectively, the calculated shear strength was 108.5 kN, which was 2.5% less than the shear strength of the RS25-0-U specimen. Therefore, it can be concluded that there is no necessity to consider the effects of UHPC and welded collars when calculating the shear strength of rubber-sleeved stud shear connectors.

**Figure 12:**
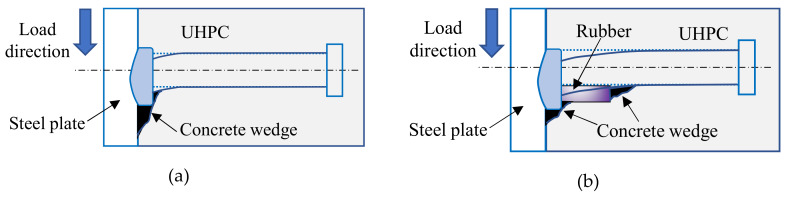
Failure modes of shear connectors in UHPC: (**a**) ordinary stud shear connectors; (**b**) rubber-sleeved stud shear connectors.

The difference between two types of shear connectors in shear strength is undoubtedly determined by the present of rubber sleeves. Figure 12 shows the states of deformation and the failure mechanisms for the shear connectors in UHPC. For an ordinary stud shear connector, the stud deformations concentrate in a region of about 10mm above the stud’s base as reported by Doinghaus et al. [33]. The compression forces in the UHPC concentrate within a compressive wedge under this region, and the weld collar carries the largest part of the ultimate load. With the present of rubber sleeves, additional bending deformation of the stud takes place in the rubber-sleeved region. The compressive wedge under the weld collar is smaller. Thus, its contribution to the shear strength of studs is smaller when the connector is failed, and the shear strength of rubber-sleeved stud shear connector is smaller.

### 3.3. Shear Stiffness

Shear stiffness is an important parameter in the design of composite structures [35]. Here, the shear stiffness is defined as the secant slope of the shear load-slip curve at the slip of 0.2 mm [35]. Table 5 lists the experimental results. The shear stiffnesses of the RS25-0-U and S-0-U specimens are 88.6 kN/mm and 235.4 kN/mm, respectively. The ratio of the two stiffnesses is 0.376. Similar to the test results in NSC [27], the shear stiffness of the connector in UHPC decreases with the height of the rubber sleeve. 

**Figure 13:**
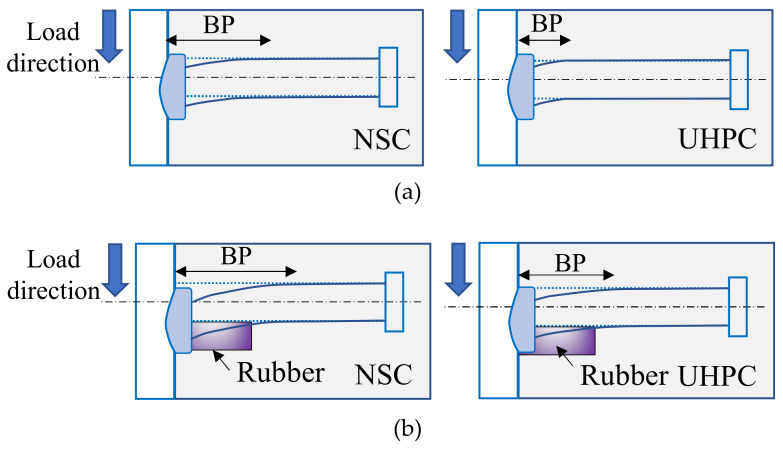
Deformation of shear connectors in NSC and UHPC: (**a**) ordinary stud shear connectors; (**b**) rubber-sleeved stud shear connectors.

It has been reported that the two shear connectors in NSC have stiffnesses of 75.3 kN/mm and 161.5 kN/mm, respectively. UHPC increased the stiffnesses of the two shear connectors by 17.7% and 45.8%, respectively. This can be explained by the higher elastic modulus of UHPC, which results in a stronger restraint to the stud deformation and a more significant stress concentration at the stud root. UHPC increases the stiffness of S shear connectors more significantly than RS shear connectors, because the contribution of the weld collar to the stiffness of the two types of shear connectors is different. As shown in Figure 13, the weld collar increases the cross-section stiffness of the stud. For both ordinary studs and rubber-sleeved studs in UHPC, the segment of the stud involved in bending deformation, the bending part (BF), is smaller than that in NSC. 

However, since the rubber-sleeved stud exhibits large deformation in the range of the rubber sleeve, there is less difference between the deformation states of studs in UHPC and NSC. As a result, the weld collar has less contribution to the increase in shear stiffness of the rubber-sleeved stud, and the increase in shear stiffness due to UHPC would decrease with the rubber sleeve height.

An analytical model based on ‘beam on elastic foundation’ theory has been established by Xu et al. [26] for calculating the shear stiffness of rubber-sleeved studs which was found to be the function of the rubber sleeve height. The ratio of the shear stiffnesses of RS and S shear connectors is calculated with Equation (2):

(2) $$\frac{k_{\mathrm{RS}}}{k_{S}}=\exp\left(-0.648\frac{h_{r}}{d_{s}}\right)$$

where *k*_RS_ and *k*_S_ are the shear stiffnesses of RS and S shear connectors; *h*_r_ is the rubber sleeve height; *d*_s_ is the diameter of the stud shank. The ratio was calculated to be 0.426 which is larger than the test result, as shown in Table 5. Obviously, the theoretical formula slightly overestimates the shear stiffness of RS shear connectors in UHPC. This is because the possible influence of weld collars was not considered in the “elastic foundation beam” model which assumes the stud to be a beam with constant cross section.

## 4. Fatigue Behavior

### 4.1. Fatigue Failure Modes

**Figure 14:**
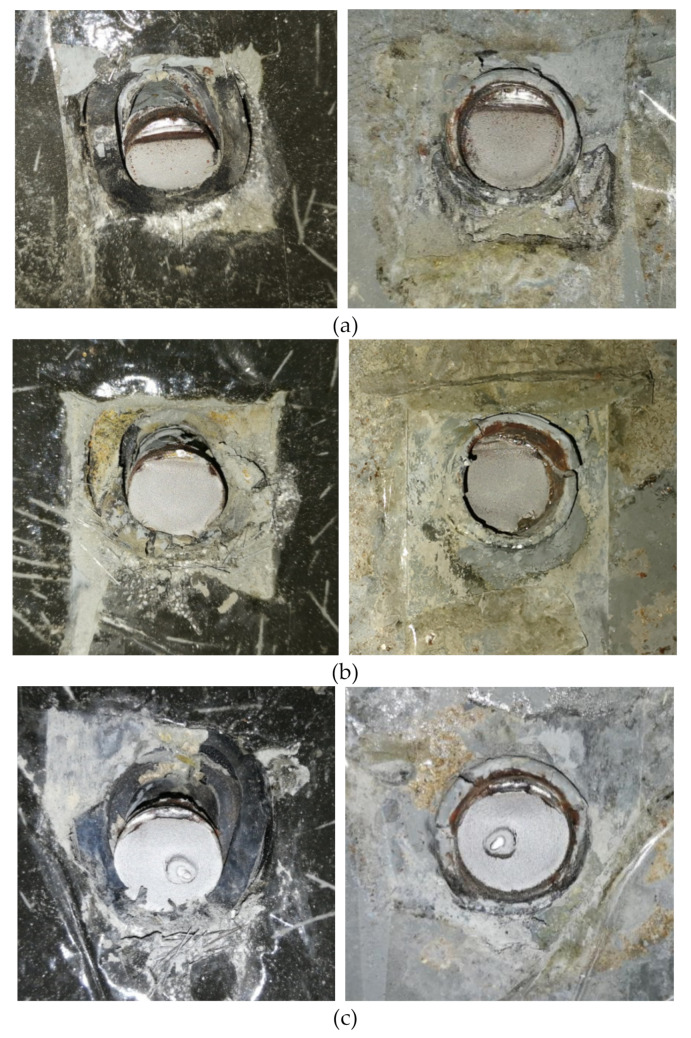
Fatigue failure modes: (**a**) RS25-50-U; (**b**) RS25-70-U; (**c**) RS25-90-U.

Figure 14 shows the state of the stud and its surrounding rubber sleeve, UHPC, and steel plate. 

The fatigue modes of all specimens in this study was close to the specimens using NSC [30]. Compared to NSC slabs in [30], cavities below the studs in the UHPC slabs were smaller. Moreover, a small amount of steel fibers peeled off from the cement matrix. The beginning of crack was believed to form at the stud shank. Then, the successive crack formed through the shank. This failure mode is similar to that of studs in NSC [30].

### 4.2. S-N Results

Table 6 summarizes the fatigue lives of specimens. Obviously, the fatigue life of the specimen (*N*_f,Exp_) decreases with the stress range. 

**Figure 15:**
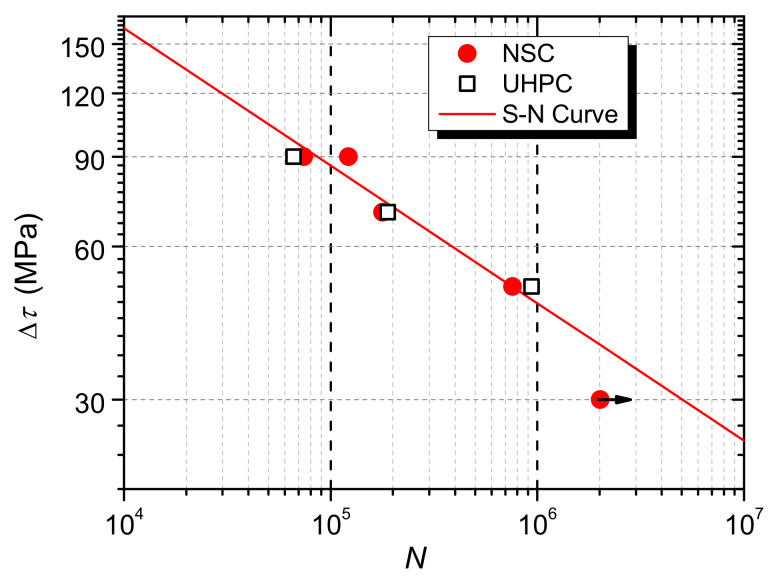
Fatigue lives of shear connectors.

The fatigue lives of shear connectors in UHPC were compared with those in NSC [30], as shown in Figure 15. 

At lower stress ranges, that is, at stress ranges of 70 MPa and 50 MPa, the fatigue life of the shear connector in UHPC is slightly greater than that in NSC, while at the stress range of 90 MPa it is slightly smaller.

S-N curves for rubber-sleeved stud shear connectors in NSC have been established by Xu et al. [30] through linear regression analysis on fatigue test data. Equation (3) is for connectors with 25mm rubber sleeves [30], and it is plotted in Figure 15. The calculation results are listed in Table 6. It can be known that the existing formula can well predict the fatigue life of the shear connector in UHPC. This further shows that UHPC has a negligible effect on the fatigue strength of the rubber-sleeved stud shear connector.

(3) logN+3.700logΔτ=12.166

where *N* is the fatigue life, and Δ*τ* is the shear stress range.

### 4.3. Results of Strain Gauges

**Figure 16:**
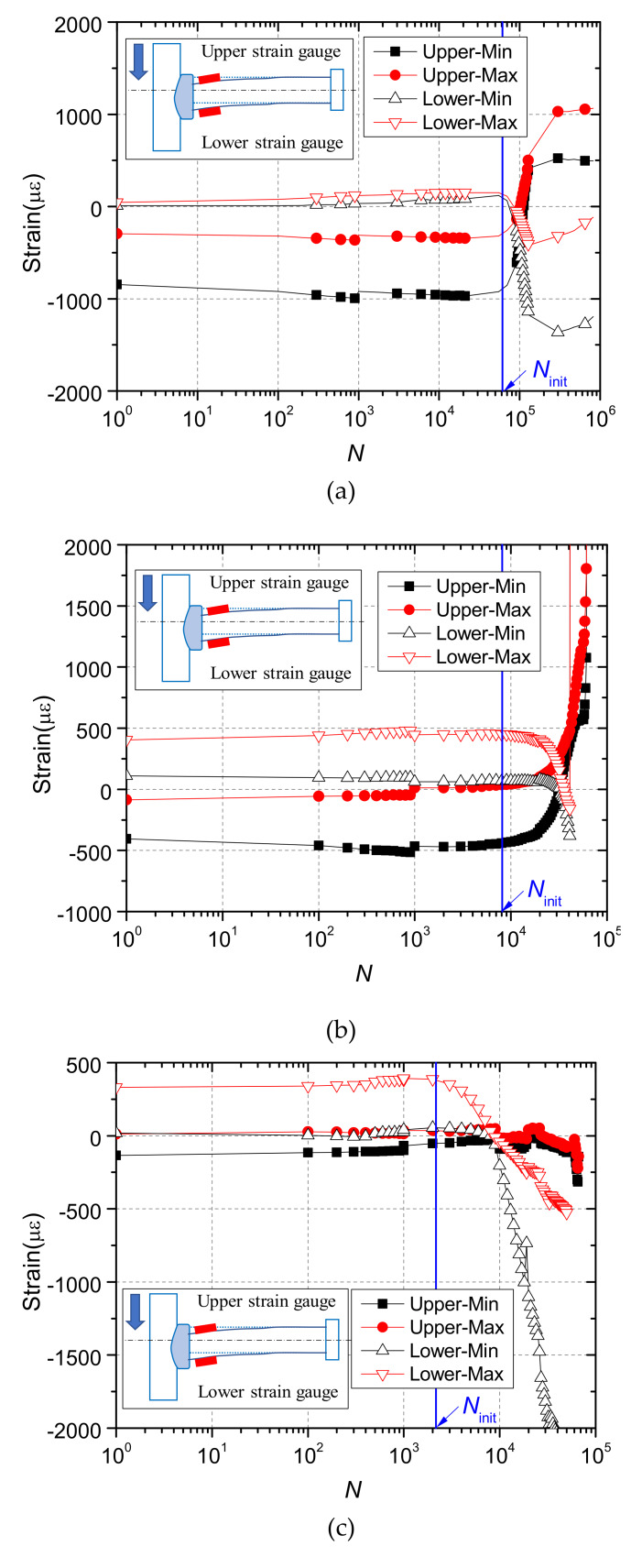
Results of strain gauges: (**a**) RS25-50-U; (**b**) RS25-70-U; (**c**) RS25-90-U.

Figure 16 plots the results of strain gauges at stud roots. In the initial stage of fatigue loading, the upper side of the stud is in compression and the lower side is in tension. Moreover, the maximum and minimum strain values remain basically unchanged. When the number of fatigue cycles is greater than a certain value, the sign of the strain starts to change. The strain in the upper side gradually becomes tensile strain, while the strain in lower side becomes compressive strain. After that, the strain increases rapidly with the number of fatigue cycles until the specimen fails.

**Figure 17:**
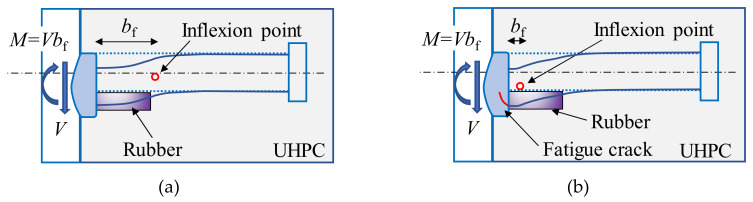
Shear mechanism of rubber-sleeved stud shear connectors: (**a**) before crack initiation; (**b**) after crack initiation.

The change in the sign of the strain was analyzed based on the shear mechanism of rubber-sleeved stud shear connectors, as shown in Figure 17. 

Due to the restraints from the steel plate and weld collar, the left end of the stud shank is almost fixed. The fixed end moment (*M*) leads to an inflexion point within the bending part of rubber-sleeved studs, which is referred to the simulation results for shear connectors with 25 mm rubber sleeves published by Xu et al. [26]. In the part to the left of the inflexion point, the upper side of the stud is in compression and the lower side is in tension, while it is opposite in the part to the right. It was reported by Xu et al. [26] that the distance from the inflexion point to the end of the stud shank (*b*_f_) increases with the rubber sleeve height. For rubber-sleeved stud shear connectors with 25 mm rubber sleeves, *b*_f_ is about 30mm. This indicates that the strain gauges are located to the left of that point at the initial stage of the fatigue test. Therefore, the test results are reasonable. With the initiation and propagation of the fatigue crack at the end of the stud shank, the area of the cracked section decreases and the stress in the section increases. Then, the cracked section gradually yields and a plastic hinge forms, which leads to a reduction in the restraint from the steel plate and weld collar to the end of the stud. As a result, the inflexion point moves towards the end of the stud, which finally results in a change in the direction of the bending moment and the sign of the strain. Moreover, the inflexion point would disappear at the final deformed state of the stud, as shown in Figure 17.

**Figure 18:**
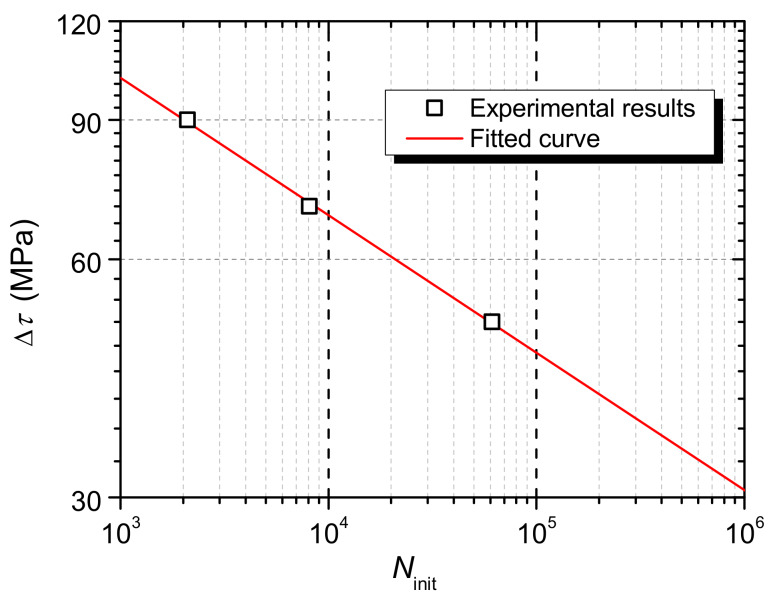
Crack initiation lives of shear connectors.

It is reasonable to assume that the change in the sign of the strain is caused by the initiation and propagation of the fatigue crack. The number of fatigue cycles when the sign of the strain starts to change is taken as the crack initiation life (*N*_init_). The results are presented in Table 6. It is clear that the crack initiation process consumes a small proportion of the fatigue life, which is about 5%. As shown in the Figure 18, the crack initiation life decreases with the stress range, and Equation (4) as a fitting formula is obtained, and the coefficient of certainty (R-Square) is 0.99.

(4) $$\log N_{\mathrm{init}}+5.757\log\Delta \tau =14.557$$

### 4.4. Results of Relative Slip and Discussion

#### 4.4.1. Results of Relative Slip

**Figure 19:**
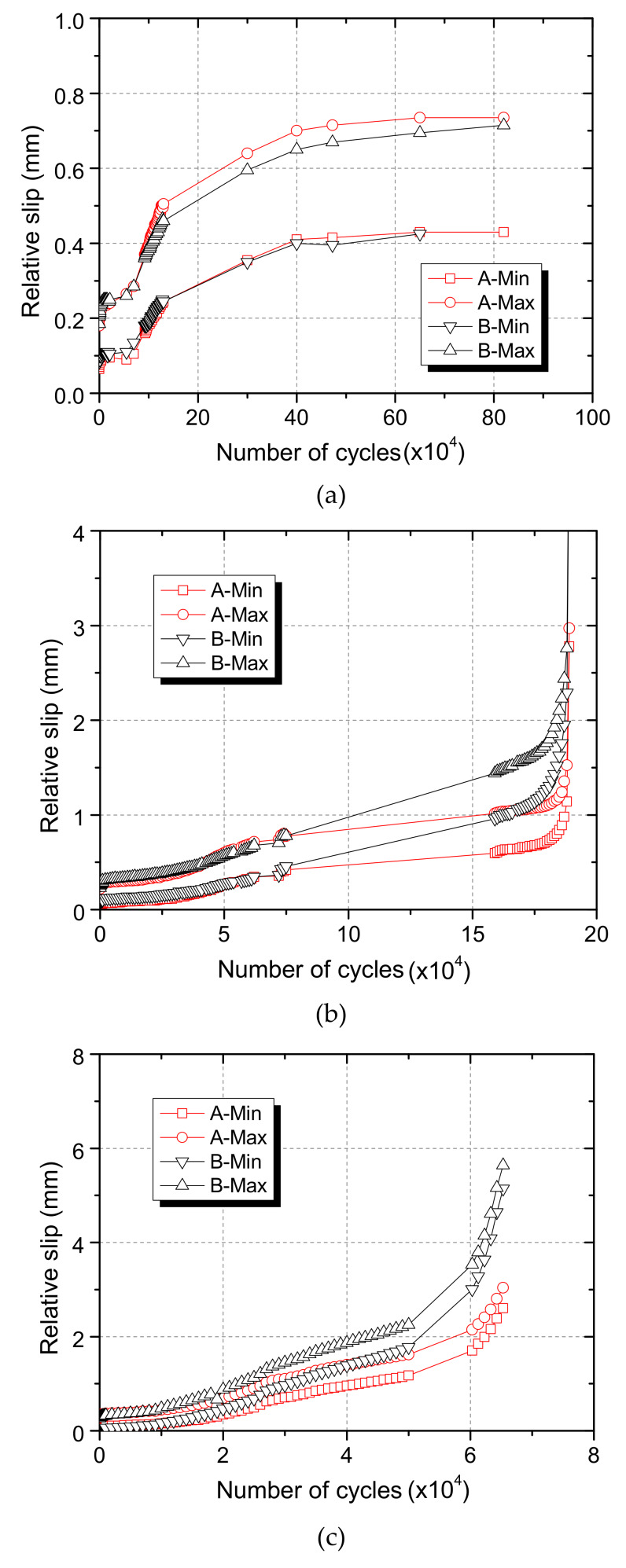
Dynamic relative slip: (**a**) RS25-50-U; (**b**) RS25-70-U; (**c**) RS25-90-U.

The measured relative slip at each side (A side and B side) of the steel component during the fatigue tests was plotted in Figure 19. The evolution laws of the dynamic relative slip were basically the same at both sides. 

#### 4.4.2. Maximum Dynamic Relative Slip Evolution Law

**Figure 20:**
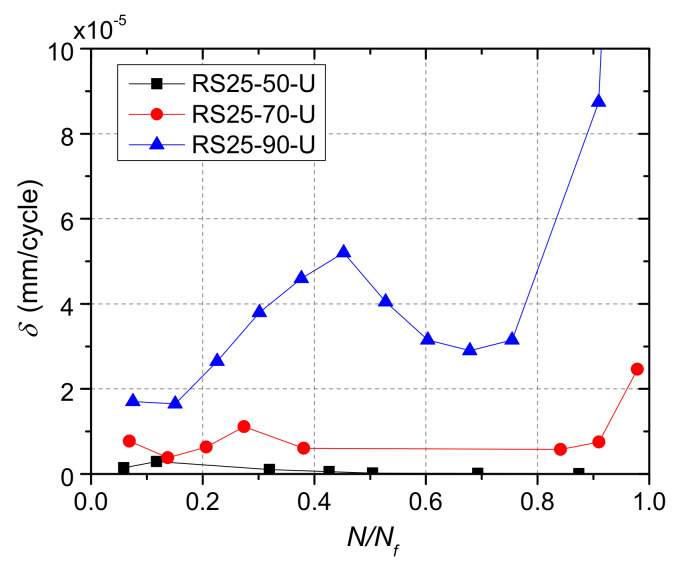
Growth rate of the maximum dynamic relative slip.

The specimens were loaded under different shear stress ranges, and the maximum dynamic relative slip of the specimen was larger for the specimen under larger shear stress range. In order to investigate the effect of shear stress range on the evolution of the maximum dynamic relative slip, the growth rate of the relative slip of each specimen was calculated and compared. The growth rate of the maximum dynamic relative slip was calculated with Equation (5)

(5) $$\delta =\frac{\Delta s_{f,\max}}{\Delta N}$$

where fatigue cycle numbers (Δ*N*), and relative slip increments (Δ*s*_f,max_). Figure 20 shows the calculated results. It was clear that though the growth rate varied during fatigue loading, but for one specimen it changed within an order of magnitude overall. Then, the average values of *δ* were calculated and listed in Table 7.

The growth law of the maximum dynamic relative slip for rubber-sleeved stud shear connectors in NSC has been studied by Xu et al. [30], and the equation for *δ* of rubber-sleeved shear connectors with 25 mm high rubber sleeves was proposed, as presented by Equation (6). The logarithmic value of *δ* was thought to be proportional to the ratio of shear force range to shear strength of the shear connectors.

(6) $$\log\delta =-7.54\;+14.55\frac{\Delta V}{V_{u}}$$

where Δ*V* is the shear force range and *V*_u_ is the static shear strength.

**Figure 21:**
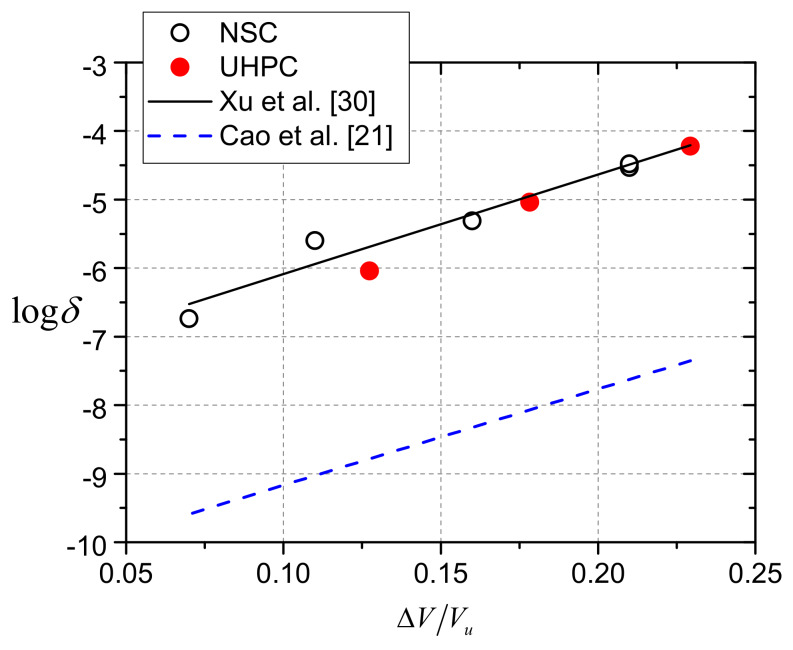
Comparison of experimental and calculated results of slip growth rate.

In Figure 21, the experimental results were compared with the results calculated from the formula originally proposed for rubber-sleeved stud shear connectors in NSC [30]. 

Obviously, the values of average growth rate of the three specimens were well predicted by the existing formula, which indicates that the connectors in UHPC show consistent fatigue characteristics with those in NSC. However, ordinary studs did not exhibit similar behavior in UHPC to that in NSC. The growth rate data of ordinary studs in UHPC reported by Cao et al. [21] showed that the rate was lower than that in NSC. It was believed that the reason was because UHPC has higher strength and stiffness than NSC and decreased the fatigue slip growth [21]. On one hand, under the same fatigue stress, UHPC has larger fatigue life and smaller stiffness degradation due to its high strength. On the other hand, studs in UHPC has smaller deformation, and the relative slip growth rate would be decreased. The corresponding equation for ordinary stud shear connectors in UHPC is Equation (7) [21].

(7) $$\log\delta =-10.574\;+14.053\frac{\Delta V}{V_{u}}$$

Obviously, in UHPC the rubber-sleeved stud shear connector has a higher growth rate than the ordinary stud shear connector, as shown in Figure 21. It has been found by Xu et al. [30] that ordinary stud shear connectors in higher strength concrete have lower the relative slip growth rate due to larger restraint provided by the concrete. However, for rubber-sleeved studs, the restraint to deformation of the stud root was significantly weakened by the rubber sleeve. As a result, compared to UHPC, rubber sleeves have a greater impact on the relative slip growth rate.

## 5. Conclusions

Five static and fatigue push-out tests were conducted to investigate the static and fatigue behavior of the rubber-sleeved stud shear connector as part of field-cast UHPC connections. The test results were analyzed to understand the effect of rubber sleeves on the behavior of shear connectors in UHPC, and several conclusions were derived.

- The slip capacities of ordinary stud and rubber-sleeved stud shear connectors are 4.6 mm and 6.9 mm, respectively. Rubber sleeves effectively improve the ductility of the shear connector in UHPC. Rubber-sleeved stud shear connectors have sufficient deformation capacity and can be used to achieve the plastic behavior of the field-cast UHPC connections in steel and concrete composite structures.
- Compared to ordinary stud shear connectors, UHPC with high compressive strength has a relatively small effect on improving the shear strength of the rubber-sleeve stud shear connector. When calculating its shear strength, it is not necessary to consider the favorable effect of UHPC and welded collars.
- The shear stiffness of the stud shear connector in UHPC decreases with the rubber sleeve height. The weld collar has less contribution to the increase in shear stiffness of the rubber-sleeved stud, and the increase in shear stiffness due to UHPC would decrease with the rubber sleeve height.
- The fatigue failure mode of the rubber-sleeve stud shear connector in UHPC is similar to that in NSC. Moreover, the S-N curve for the rubber-sleeved stud shear connector in NSC can well predict the fatigue life of the shear connector in UHPC.
- The results of strain gauges at stud roots were obtained. The change in the sign of the strain is supposed to be caused by the initiation and propagation of the fatigue crack. By taking the number of fatigue cycles when the sign of the strain starts to change as the crack initiation life, it was found that the crack initiation process consumes a small proportion of the fatigue life, which is about 5%. The crack initiation life decreases with the stress range, and a fitting formula is obtained.
- The evolution law of dynamic relative slip of rubber-sleeved stud shear connectors in UHPC are similar to those in NSC. Though UHPC improves the restraint to the stud deformation, the influence of rubber sleeves is still decisive in determining the fatigue behavior of rubber-sleeve stud shear connectors.

Rubber-sleeved stud shear connectors as part of field-cast UHPC connections in steel and concrete composite bridges have sufficient deformation capacity, which will promote the application of UHPC as the filling material for the full-depth precast concrete deck system. The static and fatigue behavior of the shear connector has been clarified in this study. However, more push-out tests and full-scale beam tests are required for establishing a design method. Moreover, finite element models have to be developed to reveal more details about the shear mechanism of rubber-sleeved stud shear connectors in UHPC.

## Figures and Tables

**Table 1:** Summary of push-out specimens.

Specimens	*h*_r_/ mm	*t*_r_/ mm	*τ*_min_/ MPa	*τ*_max_/ MPa	Δ*τ*/ MPa	*V*_min_/ kN	*V*_max_/ kN	Δ*V*/ kN	*R*	*f*/ Hz
S-0-U	—	—	—	—	—	—	—	—	—	—
RS25-0-U	25	5	—	—	—	—	—	—	—	—
RS25-50-U	25	5	12.0	62.0	50.0	3.4	17.6	14.2	0.19	4
RS25-70-U	25	5	12.0	82.0	70.0	3.4	23.2	19.8	0.15	4
RS25-90-U	25	5	12.0	102.0	90.0	3.4	28.9	25.5	0.12	4

Note: *t*_r_ is the thickness of rubber sleeves; *h*_r_ is the height of rubber sleeves; *τ*_min_ is the minimum shear stress; *τ*_max_ is the maximum shear stress; Δ*τ* is the shear stress range; *V*_min_ is the minimum fatigue load; *V*_max_ is the maximum fatigue load; Δ*V* is the fatigue load range; *R* is the stress ratio which is (*τ*_min_ /*τ*_max_); *f* is the loading frequency.

**Table 2:** Mix proportions of normal strength concrete (NSC) (kg/m^3^).

Cement	Water	Sand	Stone	Water-Reducing Agent
605.7	202.9	545.1	1090.2	6.1

**Table 3:** Material properties of NSC (MPa).

Cubic Compressive Strength	Prism Compressive Strength	Tensile Splitting Strength
51.35	38.78	3.33

**Table 4:** Material properties of NR45° natural rubber.

Hardness (Shore A)	Elongation (%)	Tensile Strength (MPa)	Brittleness Temperature (°C)	Modulus of Elasticity (MPa)
45	404	≥18	−40	7.89

**Table 5:** Results of static tests on studs in UHPC.

Specimen	Test Results			Calculated Results	
	Shear Strength (kN)	Shear Stiffness (kN/mm)	Ratio of the Stiffnesses	*η*_wc_ When *f*_c_’ = 9 9.9MPa	Ratio of the Stiffnesses
S-0-U	139.8	235.4	0.376	2.27	0.426
RS25-0-U	111.3	88.6		0.21	

**Table 6:** Fatigue tests results.

Specimens	Δ*τ*/ MPa	*τ*_min_/ MPa	*τ*_max_/ MPa	*R*	*N*_init,Exp._/ ×10^3^ Cycles	*N*_f,Exp._/ ×10^3^ Cycles	$\frac{N_{\mathrm{init},\mathrm{Exp}.}}{N_{f,\mathrm{Exp}.}}\;$	*N*_f,Cal._/ kN
RS25-50-U	50.0	12.0	62.0	0.19	61.2	938.0	0.065	758.2
RS25-70-U	70.0	12.0	82.0	0.15	8.1	189.0	0.043	218.3
RS25-90-U	90.0	12.0	102.0	0.12	2.1	66.3	0.032	86.2

**Table 7:** Experimental results of average growth rate.

Specimens	Δ*V*/ kN	*V*_u_/ kN	Δ*V*/*V*_u_	*δ*/ mm/cycle	log*δ*
RS25-50-U	14.18	111.3	0.11	9.130 × 10^−7^	−6.040
RS25-70-U	19.85	111.3	0.16	9.125 × 10^−6^	−5.040
RS25-90-U	25.53	111.3	0.21	5.966 × 10^−5^	−4.224
